# The Psychological Impact of the COVID-19 Pandemic on Pregnant Women

**DOI:** 10.3390/healthcare9060725

**Published:** 2021-06-12

**Authors:** Ruxandra-Gabriela Cigăran, Radu Botezatu, Elma-Maria Mînecan, Corina Gică, Anca Maria Panaitescu, Gheorghe Peltecu, Nicolae Gică

**Affiliations:** 1 Department of Obstetrics and Gynecology, Filantropia Clinical Hospital, 011171 Bucharest, Romania; radu.botezatu@umfcd.ro (R.B.); corina.gica@drd.umfcd.ro (C.G.); anca.panaitescu@umfcd.ro (A.M.P.); gheorghe.peltecu@umfcd.ro (G.P.); gica.nicolae@umfcd.ro (N.G.); 2 Department of Obstetrics and Gynecology, Carol Davila University of Medicine and Pharmacy, 020021 Bucharest, Romania; 3 Department of Pediatric Psychiatry, Prof. Dr. Al. Obregia Psychiatric Clinical Hospital, 041735 Bucharest, Romania; minecan.elma@yahoo.com

**Keywords:** pregnancy, pandemic, healthcare, COVID-19, mental health

## Abstract

Background: The COVID-19 pandemic has meant significant precautions and changes in delivering healthcare services. The aim of the study was to explore the lifestyle changes of pregnant women during the COVID-19 pandemic in Romania, the changes in prenatal care and delivery during the pandemic and the psychological impact on women and to determine how healthcare providers can help them to overcome this period. Methods: A cross-sectional survey was conducted anonymously and distributed among pregnancy-related groups from Romania, recruiting 559 study participants, between May and October 2020. A total of 559 pregnant women completed an online 26-item questionnaire but we only validated 557 responses for study. The survey included basic demographic questions, pregnancy-related questions, questions regarding the pregnant women’s lifestyle changes during the pandemic and their perception of the COVID-19 pandemic and questions which evaluated the impact of the pandemic on prenatal care and delivery in Romania. Results: The pandemic restrictions affected women’s routine activities regarding professional, familial and social life. Therefore, for pregnant women who were emotionally vulnerable, these restrictions had a great impact on their mental health. The majority of the study participants (78.8%, N = 439) were emotionally affected by the pandemic. The fear related to the possibility of having their pregnancy affected by the virus was dominant in the group (45.8%). A high number of women (69.5%) felt safe when they accessed health services, but private hospitals were considered safer (53.1%) compared to public hospitals (14.4%). The majority of participants (53.7%) used to have prenatal care in a private healthcare system. During the pandemic, preventive measures were associated with low confidence in the healthcare system. Of the total group of participants, 123 women (22.1%) gave birth during the pandemic. Of these, a majority of the study participants considered that it was very difficult for them to cope without their partner during the hospitalization and labor period. Conclusions: The main anxiety of pregnant women were related to threats to their lives and their baby’s health because of the uncertainty caused by pandemic. It is important to know that the restrictions and the changes in maternity care had a negative impact on them. This conclusion must be taken into account when preventive measures will be decided for helping them to get through such a period. Additionally, psychological support will be essential for improving the mental health of pregnant women and for preventing a negative outcome of the pregnancy. These feelings must be taken into account when preventive measures will be established during pandemic and a psychological support will be essential for improving their mental health for preventing negative outcome of pregnancy.

## 1. Introduction

On 11 March 2020, The World Health Organization (WHO) declared the Coronavirus disease (COVID-19) a pandemic [1]. COVID-19 has been considered a major worldwide health crisis in the 21st century and has severely affected the economy, global healthcare systems and the quality of life of different population groups [2,3]. The virus was confirmed in Romania on 26 February 2020, when the first case was detected. To date, the National Institute of Public Health has reported around 1,070,000 cases, 1,030,000 recoveries and 30,100 COVID-19-related deaths. Romania’s population is 19,122,651. As of 14 March 2021, the average age of cases is 48. The average age of deaths is significantly higher, at 71 [4]. 

To limit the spread of the SARS-CoV-2 infection, the Romanian government had implemented intensive health precautions including quarantine, wearing masks and social distancing. A national lockdown was declared in the Romania and all non-essential travel and contact with other individuals, outside a person’s home environment, was forbidden. In Romania, significant changes were adopted in hospitals such as social distancing measures, personal protective equipment for medical staff, postponement of non-essential surgery and changes in healthcare services and protocols [2,3,5,6,7]. In prenatal care, “face-to-face” antenatal visits were reduced and telemedicine started to be used. Restrictions were imposed on visits during hospitalization and on birthing partner presence [7].

Additionally, at the beginning of the outbreak, much remained unknown, from the means of transmission and symptoms, to the risk of developing complications related to COVID-19 and treatment [2,3,5,6]. Moreover, data on the pregnant women’s reaction to SARS-CoV-2 infection and fetal transmission were limited. Social media described the virus as a “killer”, so the fear of death was inevitable among people [3,8,9].

All of these changes, public panic and flow of misinformation may have exacerbated the level of stress and anxiety, especially in the vulnerable populations, which includes pregnant women. The quarantine period was associated with loneliness, boredom and uncertainty [8,9,10].

Pregnancy represents a vulnerable period regarding the psychological and emotional state of women and it has been reported that pregnant women go through anxiety and depressive symptoms compared to non-pregnant women [11,12,13]. Associating the effects of the with pregnancy, this group may be particularly at risk of psychological distress.

Stress and depression during pregnancy may be associated with some adverse obstetrical outcomes including maternal disorders (gestational diabetes, hypertension), abortion, premature birth, lower birth weight or even fetal death [12,13,14]. To date, a large number of studies have shown that stress during pregnancy can have a negative influence on pregnancy outcome and as well as on the physiological development of children and their behavior [12,13,15,16]. Lobe M. et al. showed that pregnancy-specific stress was directly associated with preterm delivery and indirectly associated with low birth weight, through its association with smoking [15].

Some studies suggested that activation of the maternal stress response and the changes in maternal endocrine and inflammatory systems play a role in the etiology of these effects on pregnancy outcome and on child development [15,16]. Davis EP et al. considered that prenatal stress can alter the course of fetal neurobiological development [16].

For these reasons, we considered it essential to explore the psychological impact of the COVID-19 pandemic on this vulnerable group, as well as to identify successful supportive strategies for helping pregnant women.

This study was designed to explore pregnant women’s perceptions of the COVID-19 pandemic and the restrictions imposed and their healthcare experiences during the pandemic. One of the purposes was to obtain insights into the changes to the healthcare system because of the pandemic, which affected pregnant women and enhanced their concerns. We hope that our findings will be used to increase the support for this vulnerable category and to improve maternity services during the pandemic.

## 2. Materials and Methods

### 2.1. Design

According to our study protocol, we established a cross-sectional survey design and we created a questionnaire for data collection. The survey was available online in Romanian language. It was conducted anonymously using Google Forms and distributed on Facebook and pregnancy-related groups and professional communities between May and October 2020.

A 26-item questionnaire was created by a team which included obstetricians and a psychiatrist. The items consisted in closed-ended questions and multiple choice. The survey included basic demographic and pregnancy related questions, regarding the pregnant women’s life changes, their perception of COVID-19 pandemic and questions which evaluated the impact of pandemic on prenatal care and delivery in Romania. The aim of the study was to explore the life changes of pregnant women, the changes in the prenatal care and delivery and the psychological impact on women during the COVID-19 pandemic in Romania.

We obtained the approval of the Ethic Council of Filantropia Hospital, Bucharest (No.38/31.03.2021).

### 2.2. Participants

All pregnant women during the COVID-19 pandemic in Romania were invited to participate in the study; the study sample consisted of pregnant women recruited via the internet who agreed to participate. A total of 559 pregnant women completed the questionnaire; however, we received only 557 valid responses. Two of them had incomplete responses and they were excluded. Data were collected online through the Google Forms platform. Before completing the questionnaires, the participants received information regarding the purpose of the study, data collection and storage methods. The participants took part in this research voluntarily and expressed their agreement to participate in the study.

In addition, the research ethics principles were respected, including the confidentiality of data and anonymity of the participants. Self-identified pregnant women were recruited via Facebook, including pregnancy-specific Facebook groups. The survey link was also shared with pregnancy-specific professional communities for distribution through their networks of pregnant women.

### 2.3. Analysis of Data

The variables were collected in an Excel database which was imported and transformed into an SPSS database. Descriptive statistics and significance tests were performed. The Chi-Square test, the corresponding corrections when the criteria were not met (Likelihood ratio, Fisher test) and the Phi and Cramer V parameters to determine the effect size were used to verify the variable associations. The results were presented numerically and graphically.

## 3. Results

### 3.1. General Aspects of Participants and Life Changes during Pandemic

**Figure 1:**
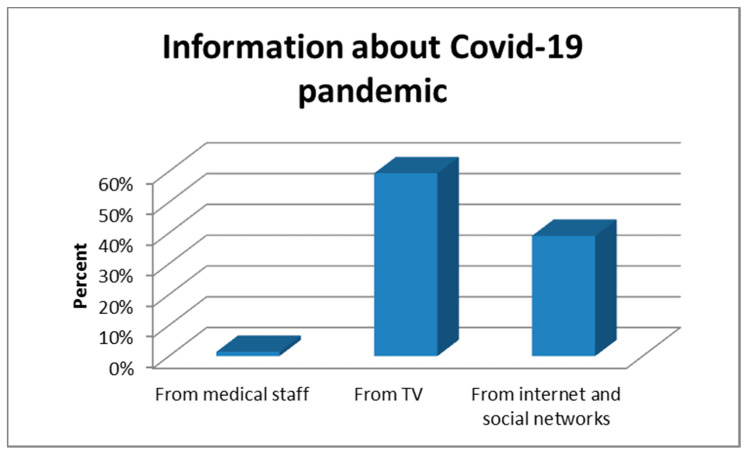
Information about COVID-19 pandemic.

In our study group, most participants found out about the COVID-19 pandemic from television (59.4%) (Figure 1).

Most women (88.5%) resided in urban areas. During the pandemic, 67.3% lived in a flat and 32.7% in a house and a proportion of 38.4% continued working from home and 28.7% were on holiday. The participants’ partners worked from home in 40.4% of cases; 39.9% continued their activity as before the pandemic, 14.4% stopped working and 5.4% were on holiday. Most women did not isolate themselves from their partners during the pandemic (94.6%), but 70.7% isolated themselves from their family, while 29.3% were not. Of all groups, 1.4% of women have continued to travel abroad since cases of COVID-19 infections have occurred in Romania and 4.3% have had contact with people who have traveled in areas affected by the pandemic.

Most of the participants (63.2%) were in the third trimester of pregnancy, 31.1% in the second trimester and 5.7% in the first trimester.

Almost all participants in the study (96.9%) stopped attending personal care locations during the pandemic and 31.6% of them were affected by this decision.

In our group, there is a statistically significant association between the attendance at personal care services during the pandemic and the form of work performed (women who continued to work accessed personal care services) and the size effect is moderate (*p* = 0.023, (df 3), (Phi/Cramer′s V = 0.148)). In addition, the use of personal care services was correlated with: the frequency of travel abroad with a moderate effect size (*p* = 0.022, (Phi/Cramer′s V = 0.154)], the contact with people who traveled in areas affected by the pandemic with moderate effect size (*p* = 0.032, (Phi/Cramer′s V = 0.117)] and the maintaining physical contact with extended family with moderate effect size (*p* = 0.012, (Phi/Cramer′s V = 0.115)). At the same time, participants from urban areas were more affected by the interdiction of access to personal care centers compared to participants from rural areas with a moderate effect size (*p* < 0.001 (df 1), (Phi/Cramer′s V = 0.154)].

To note that 55.5% of the pregnant women believed that the pandemic did not influence their couple’s relationship, 29.1% considered that their relationship improved and only 15.4% appreciated that their relationship has been deteriorated.

There is an association between the couple’s relationship and the form of work adopted, so women who continued to work, especially from home, experienced a more severe deterioration of the relationship than women who stopped working with moderate effect size (*p* = 0.037 (df 6), (Phi/Cramer′s V = 0.155/0.110)). Statistically, the couple’s relationship also deteriorated significantly even when the partner worked from home, with a moderate effect size (*p* < 0.001 (df 6), (Phi/Cramer′s V = 0.218/0.154)]. In conclusion, work from home increases the tensions between partners.

**Figure 2:**
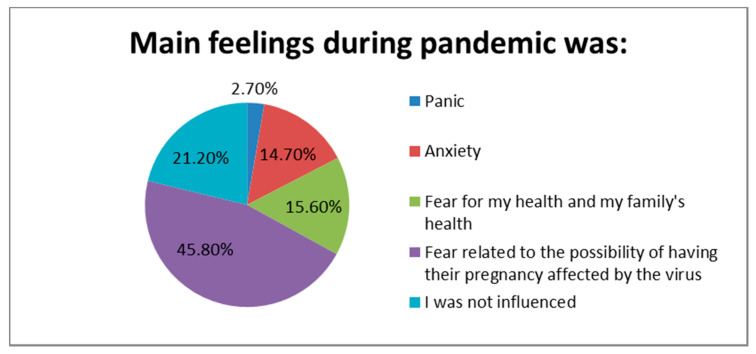
Main feelings during pandemic.

The majority of the study participants (78.8%, N = 439) were emotionally affected by the pandemic. The fear related to the possibility of having their pregnancy affected by the virus was dominant into the group (45.8%) (Figure 2).

We found statistically significant evidence that pregnant women who stopped working reported more frequent panic and anxiety and those who continued their work in the same conditions felt more frequent fear related to the possibility of having their pregnancy affected by the virus (*p* = 0.005, moderate effect size (Phi/Cramer′s V = 0.217/0.125)].

### 3.2. Health Service Access during Pandemic

**Figure 3:**
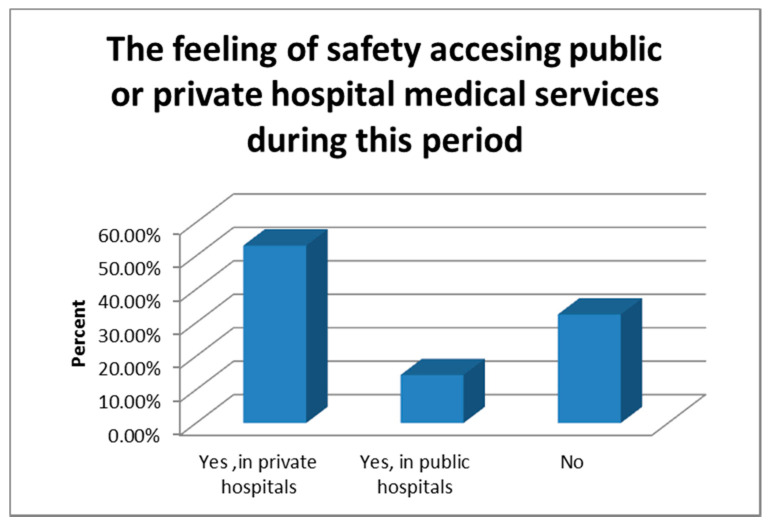
The safety accessing health service during pandemic.

The majority of women (69.5%) felt safe when they accessed health services and private hospitals were considered safer (53.1%) compared to public hospitals (14.4%) (Figure 3). The majority of participants (53.7%) used to have prenatal care in private health system. Telemedicine was embraced for medical consultations in 19% of cases.

Regarding the attitude of the medical staff, 32.3% of the participants perceived them as more dedicated and 52.2% did not perceive any difference in the medical staff behavior. Only 15.4% considered the medical staff less involved.

The presence of fears was significantly associated with less confidence in the health system compared to people who stated that their emotional condition was not influenced by the pandemic, with a moderate effect size (*p* < 0.001 (df 4), (Phi/Cramer′s V = 0.220)).

**Figure 4:**
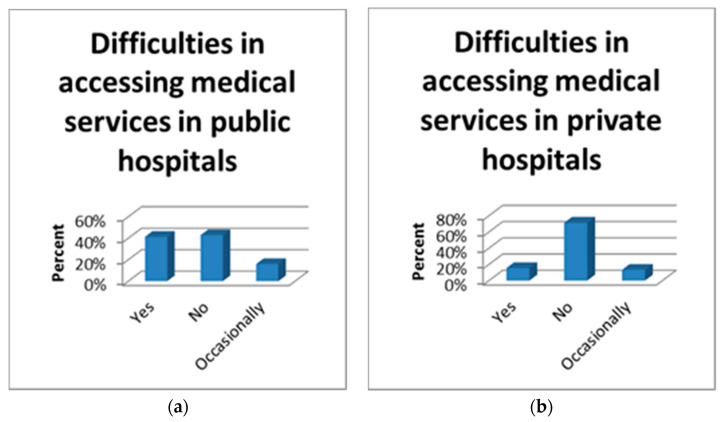
Difficulties in accessing medical services in public (**a**) and private hospitals (**b**).

To note that 77.9% of the participants continued to do prenatal investigations normally, 16.2% used only telemedicine and 5.9% gave up pregnancy care. Of all the group of pregnant women, 57.1% encountered difficulties in accessing medical services in public hospitals compared to 28.7% who had difficulties in accessing medical services in private hospitals (Figure 4). At the same time, a majority of the study participants (73.4%, N = 409) stated that they were not refused at the health units because they did not represent a medical emergency.

Among group participants, it is observed that the perception of safety accessing the health services decreases with the progress of the pregnancy and the effect size was moderate (*p* = 0.008 (df 2), (Phi/Cramer′s V = 0.131)). Access to public or private health services is associated with the activity of the participants; thus, the participants who stopped working accessed the public system more frequently than the participants who continued to work with moderate effect size (*p* = 0.002 (df 3), (Phi/Cramer′s V = 0.165)). The same tendency to attend public or private hospitals is observed depending on the partner’s work with moderate effect size (*p* < 0.001 (df 3), (Phi/Cramer′s V = 0.220)). Pregnant women felt less safe in the public sector than in the private sector with moderate effect size (*p* < 0.001 (df 1), (Phi/Cramer′s V = 0.201)). On contrary, the women with advanced pregnancy preferred the antenatal care in public hospitals and the effect size was moderate (*p* < 0.001 (df 2), (Phi/Cramer′s V = 0.206)).

Accessing telemedicine is associated with the frequency of fears, women who responded that they feel fears preferred telemedicine services in a higher proportion with moderate effect size (*p* = 0.009 (df 4), (Phi/Cramer′s V = 0.147)).

In the study group, women who stopped prenatal care were correlated with: women who stopped working (*p* = 0.036 (df 6), moderate effect size (Phi/Cramer′s V = 0.164/0.116)]; women whose couple’s relationship was deteriorated [*p* = 0.019 (df 8), moderate effect size (Phi/Cramer′s V = 0.201/0.142)]; women who felt fears/anxiety (*p* = 0.018 (df 8), moderate effect size (Phi/Cramer′s V = 0.183/0.130)); women who did not feel safe accessing health services (*p* < 0.001 (df 4), moderate effect size (Phi/Cramer′s V = 0.243/0.172)); women who perceived the medical staff less dedicated (*p* = 0.041 (df 4), weak-moderate effect size (Phi/Cramer′s V = 0.139/0.098)); women who had antenatal care in public hospitals (*p* < 0.001 (df 2), moderate effect size (Phi/Cramer′s V = 0.183)).

Women with negative emotional conditions (panic, anxiety, fear for their life/pregnancy or family) stated, statistically significantly, that their medical consultations were refused by medical staff because they did not have an emergency compared to women who considered that the pandemic did not affect their mental condition (*p* = 0.008 (df 4), moderate effect size (Phi/Cramer′s V = 0.156)).

Participants who were refused consultations had a higher degree of insecurity regarding medical services (*p* = 0.003 (df 1), moderate effect size (Phi/Cramer′s V = 0.129)); they also stated to a greater extent that the medical staff were less dedicated (*p* < 0.001 (df 2), moderate effect size (Phi/Cramer′s V = 0.204)). The rejection rate was higher in public hospitals (*p* < 0.001 (df 1), moderate effect size (Phi/Cramer′s V = 0.183)). In addition, the refusal of consultation was associated with an increase rate of giving up pregnancy care and an increase request for telemedicine (*p* < 0.001 (df 2), strong effect size (Phi/Cramer′s V = 0.323)).

Difficulties in accessing medical services in public hospitals are associated with: an increased level of insecurity, subjective perception regarding the public health system (*p* = 0.005 (df 2), moderate effect size (Phi/Cramer′s V = 0.139)); an increased demand for telemedicine services (*p* = 0.003 (df 2), moderate effect size (Phi/Cramer′s V = 0.144)) and higher rates of giving up pregnancy care (*p* = 0.033 (df 4), weak-moderate effect size (Phi/Cramer′s V = 0.138/0.097)).

**Figure 5:**
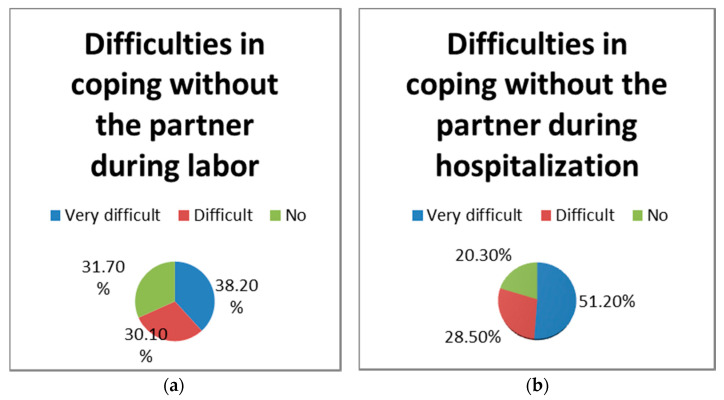
Perception on not having their partner during labor (**a**) or hospitalization (**b**).

In our group, 123 women (22.1%) gave birth during the pandemic. Of these, 68.3% considered that it was very difficult and difficult for them to cope without the partner during labor. For 79.7% of them, it was very difficult and difficult without the partner during the hospitalization period (Figure 5). In the delivery group, 73.2% of women answered that the pandemic restrictions did not affect the relationship with the baby during hospitalization.

The negative perception of not having their partner during labor is statistically associated with negative emotional conditions, so those who reported fears perceived the labor more difficult without their partner than those who said they were not emotionally influenced by the pandemic (*p* = 0.036 (df 8), moderate-strong effect size (Phi/Cramer′s V = 0.347/0.246)); it is also significantly associated with higher levels of insecurity in public and private hospitals (*p* = 0.005 (df 4), moderate-strong effect size (Phi/Cramer′s V = 0.346/0.244)); the negative perception about medical staff (*p* = 0.001 (df 4), moderate-strong effect size (Phi/Cramer′s V = 0.394/278)) and the participants who use only telemedicine (*p* = 0.005 (df 4), moderate-strong effect size (Phi/Cramer′s V = 0.331/234)).

The degree of difficulty perceived of not having their partner during hospitalization was statistically significant associated with: the negative perception of health system (*p* = 0.038 (df 4), moderate effect size (Phi/Cramer′s V = 0.229)); the negative perception of medical staff (*p* = 0.001 (df 4), moderate-strong effect size (Phi/Cramer′s V = 0.363/257)); the pregnant women who use only telemedicine (*p* = 0.025 (df 4), moderate effect size (Phi/Cramer′s V = 0.279/0.197)); the difficulties encountered in accessing medical services in public hospitals (*p* = 0.027 (df 4), moderate-strong effect size (Phi/Cramer′s V = 0.308/218)).

It was found that there is a significant association between the negative perception of relationship between mother and baby during hospitalization and the level of insecurity in public or private hospitals (*p* = 0.024, moderate effect size (Phi/Cramer′s V = 0.214)); the negative perception of medical staff (*p* = 0.001 (df 2), strong effect size (Phi/Cramer′s V = 0.347)); the pregnancy care in a private hospital (*p* = 0.044 (df 1), moderate effect size (Phi/Cramer′s V = 0.182)); the negative perception of not having the partner during labor (*p* <0.001 (df 2), strong effect size (Phi/Cramer′s V = 0.364)); the negative perception of not having the partner during hospitalization (*p* = 0.014 (df 2), moderate effect size (Phi/Cramer′s V = 0.264)).

## 4. Discussion

It is already known that previous epidemics had a negative psychological impact on the general population and misinformation increased the anxiety [17,18]. The impact of the COVID-19 pandemic on general population is already known to be negative [19,20]. This period has negatively influenced the healthcare systems, people’s social lives and the world economy [2,3].

The research regarding Sars-Cov-2 infection, the disease evolution and its treatment is still ongoing and most information are unclear, pregnancy could be especially affected by the infection. Therefore, the negative feelings are even more understandable and expected, mainly among pregnant women groups. Pregnancy is known as a vulnerable period for women, frequently associated with depression and anxiety, which can have an important impact on pregnancy outcome [11,12,13,14]. All pandemic changes mean loneliness, anxiety or depression [19,20].

During the COVID-19 pandemic, pregnant women have experienced life changes because of restrictive measures and deterioration of social life, concerns about contracting the virus and threats of Sars-Cov-2 infection to their own life and the baby’s life, the fear of not having the proper prenatal care because of the changes in healthcare services and protocols [7,8,9,21].

There are already some findings that revealed an important prevalence of depression and anxiety among pregnant women. For example, a study from China reported a 34.2% prevalence of depression [9] and a Canadian one with 1987 participants showed depressive symptoms in 37% women and anxiety symptoms in 56.6% [21].

In addition, in our study, preventive restrictions during the pandemic involved life changes for pregnant women and they were associated with negative feelings. Most of participants isolated themselves from family and friends, stopped some routine activities (e.g., using personal care services) or avoided international travels. We observed that attention to self-image is correlated with the level of social exposure. Thus, the more socially connected women are, the more likely they are interested to continue their personal care rituals. Therefore, these women were more affected by restrictions.

Among our participants, these who stopped working reported more frequent panic and anxiety and those who continued their work in the same conditions felt fear more frequently related to the possibility of having their pregnancy affected by the virus.

We already know from previous studies that physical activity and social support reduce the risk of depression during pregnancy [22,23,24]. Our findings confirm that pregnant women who remained active, regarding professional or social life during pandemic, experienced less negative emotions.

Based on current data, pregnant women in third trimester and those with other pathologies related to pregnancy or not are more likely to develop COVID-19 symptoms due to specific physiology and immunity of pregnancy [25,26]. In the literature, the association between Sars-Cov-2 infection and teratogenicity and pregnancy complications such as preterm birth, intrauterine fetal growth restriction or miscarriage, is not described, even if it was suggested a vertical transmission of infection [27,28,29,30,31]. Even so, the limited data did not reassure the women that they and their babies are out of danger.

Another aspect which increases pregnant women’s anxiety was represented by preventive measures adopted in hospitals [2,3,4,5,6,7]; the way of prenatal care changed [7,25,26]. In addition, in Romania, some maternities were assigned for “COVID patients only” and some women needed to have prenatal care in other hospitals, the prenatal scans were reduced, telemedicine was adopted and visits during hospitalization and birthing partner presence were not allowed. We consider that women avoided the hospital or preferred the private system because of the restrictions, the fear of contracting the virus and for the shortening of waiting time due to the congestion of public hospitals in pandemic conditions.

Chmielewska et al. suggest that the pandemic has also indirect effects on pregnancy outcome due to national lockdowns, changes in healthcare system and fear of attending maternity care services. They have shown an increased maternal mortality and morbidity and stillbirth during the pandemic compared to before the pandemic [3].

According to our results, emotional aspects are interconnected, life changes during the pandemic such as changes in routine activities (personal care, job, social life), deterioration of the couple’s relationship and presence of concerns associated with low confidence in the health system. In addition, the subjective emotional conditions influence the perception of other people’s behavior; thus, the negative life changes were associated with a negative perception of the medical staff. Of course, the objectification of a lower dedication of the medical staff can influence the fear and confidence in the medical system.

Multiple studies suggest that social isolation, fear of contracting the virus and its adverse effects on pregnancy outcome, insufficient maternity care and flow of misinformation are responsible for depression symptoms and anxiety symptoms during the COVID-19 pandemic [7,8,9,10,21].

The majority of pregnant women from our study continued to do prenatal investigations as recommended by professionals and missing of antenatal care was rare and it happened more frequent in the subgroup of women who had antenatal care in public hospital. The costs of pregnancy investigations, probably because of the difficult access to public health system, may affect the further care when financial security is threatened. Another important aspect in maintaining the antenatal care is the support of the partner; thus, it was observed that where the couples’ relationship was deteriorated, the rate of giving up the pregnancy care was increased. The presence of fear among pregnant women has been associated with the abandonment of the pregnancy care and the lack of care may exacerbate fears, aspects that will be clarified in another study.

Lebel C. et al. highlighted that social support from partner, family or friends is imperative; it reduces psychological distress among pregnant women during this period [21].

It is known that mood disorders increase the risk of somatization; it also exacerbates pain [32]. A plausible explanation for the fact that fearful women had the perception of consultation refusal by medical staff consecutive to failure to meet emergency criteria may be due to the symptoms which were based on emotional feelings to a greater extent than to an organic medical problem. Even if the emergency criteria were not met in these women, the suffering caused by psychological processes was real and possibly intense, requiring specialized intervention and psychological counseling.

In our group, the women with negative emotional conditions had a negative perception of not having their partner by their side during labor or hospitalization and, overall, a negative perception of health system. At the same time, these women also had a negative perception of the relationship between mother and baby during hospitalization.

This study raises the increasing need for a multidisciplinary approach to pregnant women, obstetric and psychological, due to the consequences of the pandemic with the new responsibility for medical staff to assure psychological comfort to pregnant women who cannot reach the emotional support of their own family.

## 5. Limitations

An important limitation of our study is that we do not have a control group and cannot compare the anxiety and stress levels of our pregnant population in the pre-pandemic period. Another limitation is the fact that the study was conducted as an online survey and researchers were not able to ask further questions to clarify the responses.

## 6. Conclusions

The results of our study confirmed that the COVID-19 pandemic changed pregnant women’s lives regarding their daily routine, work or social and family life. These are associated with more concerns about their health and pregnancy outcome. This is important as they are more vulnerable regarding emotional features than the general population. Other aspects which increased negative feelings among pregnant women were the changes of the health system and the limited information about SARS-CoV-2 infection and pregnancy.

The more that changes in women’s lives because of pandemic happened, the more that negative emotional impact was observed among our study participants. At the same time, negative feelings were increased by new measures implemented in hospitals. Therefore, a negative perception of health services and medical staff was noted and this was correlated, in the same situations, with the abandonment of pregnancy care.

We can conclude that the main worries of pregnant women are related to threats to their lives and their baby’s health because of the unknown causes of the pandemic. It is important to know that the changes in maternity care have a negative impact on them and they need more support to pass over this period. Considering our findings, the negative feelings of women because of restrictions, especially those related to pregnancy care, should be taken into consideration when preventive measures are established during pandemic. In addition, a psychological support will be essential for improving the mental health of pregnant women during pandemic for preventing negative outcomes of pregnancy and long-term adverse effects on mothers and babies.

## References

[1] Cucinotta D., Vanelli M. (2020). WHO Declares COVID-19 a Pandemic. Acta Biomed..

[2] Bellato V., Konishi T., Pellino G., An Y., Piciocchi A., Sensi B., Siragusa L., Khanna K., Pirozzi B.M., Franceschilli M. (2020). Screening policies, preventive measures and in-hospital infection of COVID-19 in global surgical practices. J. Glob. Health.

[3] Chmielewska B., Barratt I., Townsend R., Kalafat E., Van der Meulen J., Gurol-Urganci I., O’Brien P., Morris E., Draycott T., Thangaratinam S. (2021). Effects of the COVID-19 pandemic on maternal and perinatal outcomes: A systematic review and meta-analysis. Lancet Glob. Health.

[4] Press Releases—Ministry of Internal Affairs. https://www.mai.gov.ro/category/comunicate-de-presa/.

[5] An Y., Bellato V., Konishi T., Pellino G., Sensi B., Siragusa L., Franceschilli M., Sica G.S., S-COVID Collaborative Group (2020). Surgeons’ fear of getting infected by COVID19: A global survey. Br. J. Surg..

[6] Bellato V., Konishi T., Pellino G., An Y., Piciocchi A., Sensi B., Siragusa L., Khanna K., Pirozzi B.M., Franceschilli M. (2020). Impact of asymptomatic COVID-19 patients in global surgical practice during the COVID-19 pandemic. Br. J. Surg..

[7] Karavadra B., Stockl A., Prosser-Snelling E., Simpson P., Morris E. (2020). Women’s perceptions of COVID-19 and their healthcare experiences: A qualitative thematic analysis of a national survey of pregnant women in the United Kingdom. BMC Pregnancy Childbirth.

[8] Ng Q.J., Koh K.M., Tagore S., Mathur M. (2020). Perception and Feelings of Antenatal Women during COVID-19 Pandemic: A Cross-Sectional Survey. Ann. Acad. Med. Singap..

[9] Wu Y., Zhang C., Liu H., Duan C., Li C., Fan J., Li H., Chen L., Xu H., Li X. (2020). Perinatal depressive and anxiety symptoms of pregnant women during the coronavirus disease 2019 outbreak in China. Am. J. Obstet. Gynecol..

[10] Ravaldi C., Ricca V., Wilson A., Homer C., Vannacci A. (2020). Previous psychopathology predicted severe COVID-19 concern, anxiety, and PTSD symptoms in pregnant women during “lockdown” in Italy. Arch. Womens Ment. Health.

[11] Bennett H.A., Einarson A., Taddio A., Koren G., Einarson T.R. (2004). Prevalence of depression during pregnancy: Systematic review. Obstet. Gynecol..

[12] Coussons-Read M.E. (2013). Effects of prenatal stress on pregnancy and human development: Mechanisms and pathways. Obstet. Med..

[13] Grigoriadis S., Graves L., Peer M., Mamisashvili L., Tomlinson G., Vigod S.N., Dennis C.L., Steiner M., Brown C., Cheung A. (2018). Maternal Anxiety During Pregnancy and the Association With Adverse Perinatal Outcomes: Systematic Review and Meta-Analysis. J. Clin. Psychiatry.

[14] Panaitescu A.M., Ciobanu A.M., Popescu M.R., Huluta I., Botezatu R., Peltecu G., Gica N. (2020). Incidence of hypertensive disorders of pregnancy in Romania. Hypertens. Pregnancy.

[15] Lobel M., Cannella D.L., Graham J.E., DeVincent C., Schneider J., Meyer B.A. (2008). Pregnancy-specific stress, prenatal health behaviors, and birth outcomes. Health Psychol..

[16] Davis E.P., Glynn L.M., Waffarn F., Sandman C.A. (2011). Prenatal maternal stress programs infant stress regulation. J. Child Psychol. Psychiatry.

[17] Brooks S.K., Webster R.K., Smith L.E., Woodland L., Wessely S., Greenberg N., Rubin G.J. (2020). The psychological impact of quarantine and how to reduce it: Rapid review of the evidence. Lancet.

[18] Rubin G.J., Harper S., Williams P.D., Öström S., Bredbere S., Amlôt R., Greenberg N. (2016). How to support staff deploying on overseas humanitarian work: A qualitative analysis of responder views about the 2014/15 West African Ebola outbreak. Eur. J. Psychotraumatol..

[19] Wang C., Pan R., Wan X., Tan Y., Xu L., Ho C.S., Ho R.C. (2020). Immediate Psychological Responses and Associated Factors during the Initial Stage of the 2019 Coronavirus Disease (COVID-19) Epidemic among the General Population in China. Int. J. Environ. Res. Public Health.

[20] Pfefferbaum B., North C.S. (2020). Mental Health and the Covid-19 Pandemic. N. Engl. J. Med..

[21] Lebel C., MacKinnon A., Bagshawe M., Tomfohr-Madsen L., Giesbrecht G. (2020). Elevated depression and anxiety symptoms among pregnant individuals during the COVID-19 pandemic. J. Affect. Disord..

[22] Demissie Z., Siega-Riz A.M., Evenson K.R., Herring A.H., Dole N., Gaynes B.N. (2011). Physical activity and depressive symptoms among pregnant women: The PIN3 study. Arch. Womens Ment. Health.

[23] Demissie Z., Siega-Riz A.M., Evenson K.R., Herring A.H., Dole N., Gaynes B.N. (2013). Physical activity during pregnancy and postpartum depressive symptoms. Midwifery.

[24] Giesbrecht G.F., Poole J.C., Letourneau N., Campbell T., Kaplan B.J., APrON Study Team (2013). The buffering effect of social support on hypothalamic-pituitary-adrenal axis function during pregnancy. Psychosom. Med..

[25] American College of Obstetricians and Gynecologists (ACOG) ACOG Patient Resource: Coronavirus (COVID-19), Pregnancy, and Breastfeeding: A Message for Patients. https://www.acog.org/womens-health/faqs/coronavirus-covid-19-pregnancy-and-breastfeeding.

[26] Royal College of Obstetricians and Gynaecologists (RCOG) RCOG Guidance: Coronavirus (COVID-19) Infection and Pregnancy. https://www.rcog.org.uk/coronavirus-pregnancy.

[27] Zeng H., Xu C., Fan J., Tang Y., Deng Q., Zhang W., Long X. (2020). Antibodies in Infants Born to Mothers With COVID-19 Pneumonia. JAMA.

[28] Chen H., Guo J., Wang C., Luo F., Yu X., Zhang W., Li J., Zhao D., Xu D., Gong Q. (2020). Clinical characteristics and intrauterine vertical transmission potential of COVID-19 infection in nine pregnant women: A retrospective review of medical records. Lancet.

[29] Zhu H., Wang L., Fang C., Peng S., Zhang L., Chang G., Xia S., Zhou W. (2020). Clinical analysis of 10 neonates born to mothers with 2019-nCoV pneumonia. Transl. Pediatr..

[30] Wong S.F., Chow K.M., Leung T.N., Ng W.F., Ng T.K., Shek C.C., Ng P.C., Lam P.W., Ho L.C., To W.W. (2004). Pregnancy and perinatal outcomes of women with severe acute respiratory syndrome. Am. J. Obstet. Gynecol..

[31] Di Mascio D., Khalil A., Saccone G., Rizzo G., Buca D., Liberati M., Vecchiet J., Nappi L., Scambia G., Berghella V. (2020). Outcome of coronavirus spectrum infections (SARS, MERS, COVID-19) during pregnancy: A systematic review and meta-analysis. Am. J. Obstet. Gynecol. MFM.

[32] Woo A.K. (2010). Depression and Anxiety in Pain. Rev. Pain.

